# Expert Consensus‐Based Technical Guidelines for Remote Robotic‐Assisted Surgery and Procedures

**DOI:** 10.1002/wjs.12653

**Published:** 2025-06-28

**Authors:** Yulun Wang, Martin Buehler, Shane Farritor, Yuman Fong, Mike Kijewski, Brian Miller, Bill Peine, Blair Whitney, Mike Yramategui, Jordana Bernard

**Affiliations:** ^1^ Sovato Santa Barbara California USA; ^2^ Johnson & Johnson Santa Clara California USA; ^3^ Virtual Incision Lincoln Nebraska USA; ^4^ Mechanical and Materials Engineering College of Engineering University of Nebraska Lincoln Nebraska USA; ^5^ Department of Surgery City of Hope Duarte California USA; ^6^ MedCrypt Solana Beach California USA; ^7^ Intuitive Surgical Sunnyvale California USA; ^8^ Medtronic Ashland Massachusetts USA

**Keywords:** healthcare telecommunications, latency, network structure, remote robotic procedure, remote robotic surgery, remote surgery, telementoring, telepresence, telesurgery

## Abstract

Remote robotic‐assisted surgery and procedures offer significant potential to enhance access to surgical expertise, optimize patient outcomes, improve healthcare efficiency, reduce costs, and increase patient and provider satisfaction, aligning with the quintuple aim. By mitigating geographic barriers, addressing surgical deserts, and reducing travel burdens for surgeons, patients, and their families, remote robotic‐assisted surgery and procedures can substantially expand access to high‐quality health care. The safe and effective implementation of remote procedures depends on the availability of robust, surgical‐grade networks, reliable connectivity, and comprehensive cybersecurity measures to protect patient data. Seamless integration among remote‐enabled robotic system interfaces, telepresence technologies, and facility‐level infrastructure is essential to ensure operational reliability and procedural success. These technical guidelines define the fundamental technical requirements to support the design, implementation, and scaling of safe, effective, and interoperable remote robotic‐assisted surgical and procedural programs. As the field evolves, guidelines will be updated to reflect technological advancements, specifications, and emerging best practices. Complementary clinical and operational practice guidelines will work synergistically with technical guidelines to support the global expansion and market adoption of remote programs.

## Graphical abstract

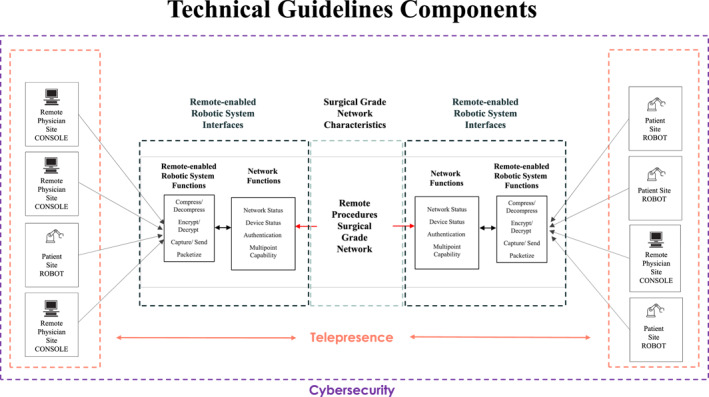

## 1 Introduction

### 1.1 The Challenge of Surgical and Procedural Access

Access to surgical care remains one of the most pressing yet unsolved public health challenges. Surgical deserts—regions lacking adequate access to surgical care—are a well‐documented global issue that affects both developed and developing countries. Today, over half of the global population lives without sufficient surgical access [1]. Even in the most advanced healthcare systems and affluent countries, access to high‐quality surgical care remains deeply inequitable, with approximately 24% of the population in high‐income countries lacking adequate surgical access [2].

In the United States, a country viewed as a leader in healthcare innovation, over one‐third of counties lack access to a single surgeon. This crisis is projected to worsen with an estimated shortage of up to 30,200 surgeons across all specialties by 2034 [3]. Although surgical expertise is often concentrated in urban centers, patients in both rural and remote areas face overwhelming barriers to care.

Multiple factors, including a shortage of skilled surgeons and interventionalists, geographic barriers, rising healthcare costs, and financial pressures on healthcare providers, drive limited access to quality surgical care. The implications of inadequate surgical access are far‐reaching: patients are left with limited, or often, no options for essential procedures, leading to costly and time‐consuming travel, delayed care, or substandard surgeries and postoperative treatment. In the developed world, these barriers drive surgical delays, create capacity bottlenecks at high‐volume hospitals, and force patients to travel long distances. In developing countries, the challenges are even more pronounced—many regions lack even the most basic surgical infrastructure.

### 1.2 Impact of Remote Procedures

remote robotic‐assisted surgery and procedures (“Remote Procedures”) represent one of the most promising advancements in modern medicine. By enabling a surgeon to operate on a patient from virtually anywhere via a remote‐enabled robotic system and high‐speed network, remote procedures have the potential to redefine surgical access by erasing geographic barriers and increasing surgical efficiencies worldwide.

Although this concept is novel in its modern implementation, its foundations are not new. In 2001, the groundbreaking Lindbergh Operation demonstrated feasibility and safety with a transatlantic remote procedure [4]. In this surgery, surgeons located in New York performed a remote robot‐assisted laparoscopic cholecystectomy on a 68‐year‐old woman in Strasbourg. The operation was successfully carried out across more than 14,000 km using a dedicated fiber‐optic communications network between the sites. Subsequently, in 2003, surgeons in Ontario, Canada, performed 22 remote robot‐assisted laparoscopic procedures between St. Joseph's Healthcare Hamilton, a teaching hospital affiliated with McMaster University, and North Bay General Hospital, a community hospital located 400 km away. These operations were facilitated using an Internet protocol virtual private network [5]. Despite these early successes, further projects were abandoned in the mid‐2000s due to high network costs, latency challenges, a lack of societal readiness, and the halted development of remote‐enabled surgical robotics. Over the past 2 decades, significant advancements in robotic‐assisted technology, telemedicine, and telecommunications, particularly the expansion of high‐speed, lower‐cost networks, and reduced latency, have reignited the potential of remote procedures as a scalable model of care [6, 7, 8].

Telemedicine has become a standard component of healthcare delivery and is typically used for consultations, even when the patient is close to their provider, as it is an effective delivery modality for both the provider and the patient. This serves as a model for remote surgery and procedures, demonstrating that geographical distance, both near and far, should not be a barrier to accessing surgical expertise.

In developed countries, remote procedures will help alleviate the growing surgeon and workforce shortage, strengthen regional hospital networks, alleviate financial strain, and create access to care for underserved communities. Remote procedures will also contribute to broader healthcare objectives, including progress toward the Quintuple Aim—a framework guiding healthcare transformation through five key pillars: (1) improved patient experience, (2) better outcomes, (3) cost efficiency, (4) clinician well‐being, and (5) health equity [9]. Remote procedure programs will drive efficiency by increasing a physician's surgical capacity, optimizing operating room utilization through load balancing, and bringing the skills of specialists to where they are needed most. For example, enabling specialists to provide remote procedures across a healthcare system or group of hospitals will help small and mid‐sized hospitals stay viable, expand patient choice, and lower costs. Beyond expanding access, remote procedures foster global collaboration, enabling physicians to share expertise, mentor colleagues, and provide advanced hands‐on surgical training.

The ripple effect in developing nations will be profound. By setting a global precedent for safe, effective, and scalable remote procedures, this innovation paves the way for expansion into underresourced regions, delivering world‐class expertise to areas where surgical care is otherwise lacking and/or nonexistent. Furthermore, following global efforts to enhance healthcare safety through technology, the World Health Organization's Global Plan of Action for Patient Safety 2021–2030 explicitly recommends investing in the digitization of end‐user health care services, such as telemedicine and telediagnosis, as a key strategy to reduce preventable harm and promote safer care delivery systems [10].

### 1.3 Value of Industry Guidelines

As healthcare rapidly embraces this transformative technology, establishing comprehensive technical guidelines is critical to ensuring patient safety and maintaining universal quality. Recognizing this need, a working group of industry leaders was formed to develop technical guidelines to inform the design and implementation of remote‐enabled robotic systems. This initiative followed industry‐recognized guidelines development processes, including consensus‐driven collaboration, broad stakeholder engagement with technical and clinical subject matter experts, and an extensive literature review.

Although primarily technical in scope, these guidelines have far‐reaching implications for the entire remote procedures ecosystem, including patient care, operational workflows, and industry alignment. By establishing clear technical guidelines, this initiative aims to support the safe and effective expansion of remote procedure programs, providing a structured framework that plays a crucial role in shaping the future of surgical care worldwide. The ultimate goal is to democratize worldwide access to safe, high‐quality, interoperable, and scalable surgical and procedural care.

### 1.4 Applying the Guidelines

The resulting *Remote Robotic‐Assisted Surgery and Procedures Technical Guidelines* define critical requirements for surgical‐grade network characteristics, cybersecurity, system interfaces, telepresence, and facility‐level considerations. The document also outlines additional key design and implementation requirements for remote‐enabled robotic systems as well as cybersecurity best practices. These guidelines do not define specifications (e.g., network latency, jitter, and bandwidth allocation) as these parameters vary based on the use case and other factors. Additionally, these specifications will be validated through the development process for defined use cases.

As remote procedures continue to evolve, these guidelines will be updated accordingly to reflect technological advancements and emerging best practices. In addition, a range of clinical, operational, and ethical considerations must be addressed to ensure patient safety and support the broader adoption of remote procedures. Complementary practice guidelines, covering issues, such as liability, licensure, credentialing, and safety protocols, will align with the technical guidelines, working together to support the safe and effective expansion of remote programs [11, 12, 13].

### 1.5 Intended Audience

These *Technical Guidelines* are intended for all stakeholders involved in designing, developing, or implementing remote‐enabled robotic systems, including the following groups:

- Medical device manufacturers and robotic companies
- Telecommunications and infrastructure providers
- Technology and cybersecurity professionals
- Healthcare organizations, hospital administrators, IT, and other professionals
- Healthcare providers and clinical teams
- Research and academic institutions
- Healthcare regulators

## 2 Scope

*The Expert Consensus‐Based Technical Guidelines for Remote Surgery and Procedures* provide a framework of fundamental technical requirements to support the design and implementation of safe, effective, interoperable, and scalable remote‐enabled robotic systems. These guidelines require collaboration among ecosystem stakeholders and should be considered in combination with other factors that contribute to safety that are beyond the scope of these guidelines.

**FIGURE 1:**
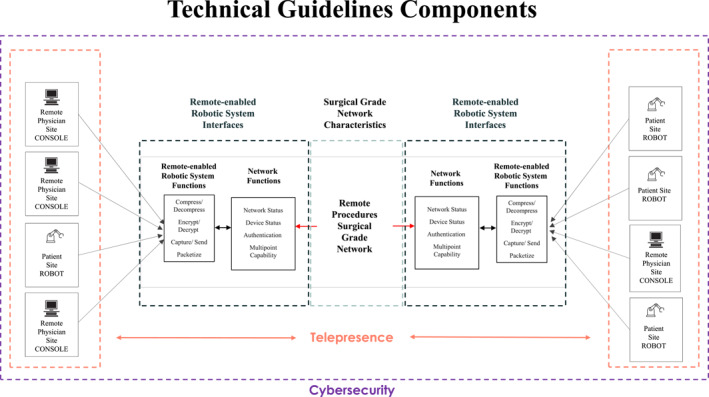
Key components addressed in technical guidelines.

The technical guidelines address these key components specific to remote procedures (e.g., robotic‐assisted surgery [RAS] and interventional delivery systems) as shown in Figure 1:

1. Operational requirements for the surgical‐grade network.
2. Cybersecurity requirements for the surgical‐grade network and remote‐enabled robotic systems.
3. Requirements for system interfaces to operate over the surgical‐grade network.
4. Requirements for telepresence in the remote physician site and the patient site procedure room.
5. Facility‐level requirements for both the remote physician site and the patient site.

The technical guidelines apply across various clinical settings (e.g., operating rooms and procedure rooms) and use cases with instrument control by the remote physician (e.g., remote minimally invasive surgery, remote tele‐mentoring, remote endoscopy, remote endovascular, remote interventions, and other diagnostic procedures).

The guidelines specify various levels of requirements using the following terminology:

- “shall” indicates a requirement
- “should” indicates a recommendation
- “may” indicates a permission

The guidelines do not address:

- Clinical personnel, clinical practice guidelines, or procedure scheduling systems.
- Remote procedures that do not involve tele‐manipulation of interventional, surgical, or diagnostic tools.
- Scenarios where the surgical robot operates autonomously without direct input or control by a remote operator.
- Broader digital health features, AI integration, or cloud connectivity beyond the immediate needs of remote robotic‐assisted surgery and procedures.

## 3 Terms, Definitions, and Abbreviations

### 3.1 Terms and Definitions

For this document, the following terms and definitions apply:

*Authorized Model or Configuration*: This refers to the authorized remote‐enabled robotic system that is permitted to connect over the surgical‐grade network. The system is defined and monitored by the robot manufacturer.

*Bandwidth*: The maximum rate of data transfer achievable over a given medium.

*Globally Unique Identifier*: An identifier assigned to each device on the surgical‐grade network, formatted following special conventions to support uniqueness within an organization and across all organizations, creating identifiers such as a Media Access Control address, globally unique identifiers defined in Request For Comments 9562, or other types of globally unique identifiers.

*Host:* A computer, device, or other endpoint that is connected to and utilizes a given network.

*Internet Protocol:* A protocol designed for interconnected packet‐switched networks, providing for transmitting data blocks (datagrams) from source to destination using fixed‐length addresses and handling fragmentation/reassembly if necessary.

*Interoperability*: The ability of two or more systems or components to exchange information and to use the information that has been exchanged.

*Jitter*: This refers to the inconsistency or variation in the time it takes for data packets to reach their destination.

*Latency*: The time measurement between when a source transmits data and the destination receives the data.

*Multipoint Capability:* Refers to a network that has two or more hosts and allows communication between any combination of hosts.

*Network Operations Team*: This refers to a team that monitors the surgical‐grade network, assesses traffic flow and capacity of all surgical‐grade network paths, and may be alerted to issues. This team receives and monitors requests, scheduled or in the moment, to ensure that all requested sessions have the required bandwidth to complete sessions without constraints.

*Network Performance Log*: This refers to a record of data collected by the surgical‐grade network related to the its operational behavior, intended to provide a detailed picture of the surgical‐grade network's functioning.

*Network Latency:* The time measurement between when a source transmits data over a network and when the source receives a response or acknowledgment from the destination over the network. This is round‐trip time (across the network and back).

*Non‐Well‐Known Ports*: A Transmission Control Protocol or User Datagram Protocol port greater than 1023 and less than or equal to 65,535.

*Other Related Devices*: Devices required for a procedure, such as imaging equipment, monitoring equipment, and other devices, that transmit data over the surgical‐grade network during the session.

*Patient Site*: The location or room of the patient undergoing a remote procedure.

*Patient Site Care Team*: The care team at the patient site supporting the remote procedure (e.g., nurses, operating room technicians, anesthesiologists, clinical engineers, and other physicians).

*Peak Load Bandwidth*: This is the maximum amount of bandwidth that a surgical‐grade network leg is expected to support at any given time, considering the combined bandwidth requirements of all projected active sessions.

*Path Maximum Transmission Unit Discovery:* A standardized technique in computer networking for determining the maximum transmission unit size on the network path.

*Real‐Time Network Communications*: This refers to network communications (e.g., not control loops) where information is transmitted and received with minimal network latency.

*Remote Physician*: The physician in a different location from the patient and care team.

*Remote Physician Site*: The location where the physician is participating in the remote procedure.

*Remote Robotic‐Assisted Surgery or Procedure*: A clinical procedure, including surgery and interventional procedures, in which a physician uses a remote‐enabled robotic system and surgical‐grade network to perform a procedure on a patient in a different location. Also known as “remote surgery,” “remote procedure,” “telesurgery,” and/or “tele‐interventions.”

*Remote‐Enabled Robotic System*: This refers to the entire system used for remote procedures, including the robotic system at the patient site, the physician's console at the remote physician site, and any hardware or software, required to enable a safe and effective procedure over a surgical‐grade network connection.

*Requested Session*: A single requested instance of a remote procedure that is performed using a surgical‐grade network—authorized remote‐enabled robotic system, with defined bandwidth requirements, and an expected time duration to ensure required bandwidth is available for the session.

*Session*: This refers to a single instance of a remote procedure performed using a remote‐enabled robotic system, encompassing the time from when the connection is established to when it is terminated.

*Session Log*: This refers to a record of data related to a specific remote procedure session intended to capture a comprehensive view of the session, including both surgical‐grade network and remote‐enabled robotic system performance.

*Surgical‐Grade Network*: A managed network with high reliability, robust security, and guaranteed quality of service, which supports the requirements of remote procedures as outlined in these guidelines.

*Surgical‐Grade Network System Interface*: Refers to the interfaces between the remote‐enabled robotic system and the surgical‐grade network to implement connection and communication between the robotic system(s) at patient site(s) and physician's console(s) at the remote location(s).

*Telepresence*: Refers to the use of audio–video technologies to create a virtual presence of the remote physician at the patient site.

*Teleoperation Latency:* The time between when the remote surgeon moves the input device to when that surgeon sees motion of the instrument on their monitor showing the video from the patient site robotic system.

*Use Case*: This refers to any remote procedure with control of the remote‐enabled robotic system by a remote physician, such as remote minimally invasive surgery, remote endoscopy, remote endovascular procedures, remote interventions, other remote diagnostic procedures, or tele‐mentoring with instrument control.

### 3.2 Abbreviations

**Table:** 

AAMI	Association for the Advancement of Medical Instrumentation
CoS	Class of Service
DAST	Dynamic Application Security Testing
DDoS	Distributed Denial‐of‐Service
FDA	Food and Drug Administration
FIPS	Federal Information Processing Standard
GDPR	General Data Protection Regulation
GUID	Globally Unique Identifier
HIPAA	Health Insurance Portability and Accountability Act
HTTP	Hypertext Transfer Protocol
ID	Identifier
IEC	International Electrotechnical Commission
IEEE	The Institute of Electrical and Electronics Engineers
IETF	Internet Engineering Task Force
IMDRF	International Medical Device Regulatory Forum
IP	Internet Protocol
ISO	International Organization for Standardization
LAN	Local Area Network
MAC	Media Access Control
MDR	Medical Device Regulation (European Union)
NIST	National Institutes of Standards and Technology
PMTUD	Path Maximum Transmission Unit Discovery
QoS	Quality of Service
RAS	Robotic‐Assisted Surgery
RFC	Request For Comments
SAST	Static Application Security Testing
SBOM	Software Bill of Materials
SSH	Secure Shell
TCP	Transmission Control Protocol
TLS	Transport Layer Security
UDP	User Datagram Protocol
WAN	Wide Area Network

## 4 Existing Standards Applicable to Remote Procedures

This section addresses existing standards relevant to remote procedures, providing a foundation for ensuring patient safety, cybersecurity, and regulatory compliance. It details key standards and provides examples across various categories, including communication and interoperability, cybersecurity and data protection, medical device quality and risk management, medical device software lifecycle, and medical device usability [14]. Section 5.2.1 provides additional details related to relevant cybersecurity regulations, guidance, and frameworks.

1. Remote procedures and related operations should adhere to applicable local and global standards to ensure patient safety, cybersecurity, and regulatory compliance as required.
2. Local and global standards that may be applicable to remote procedures include, but are not limited to, the standards listed in Table 1.

**TABLE 1:** Examples of existing standards that may be applicable to remote procedures.

Category	Standard	Region
Communication and interoperability	IEEE/UL 2933:2024	Global
Cybersecurity and data protection	ISO/IEC 27001:2022+A1:2024	Global
	ISO/IEC 19790:2012	Global
	ISO/IEC 24759:2017	Global
	ISO 80001‐1	Global
	IEC 81001‐5‐1:2021	Global
	IEC 62443‐3‐2	Global
	IEC 62443‐3‐1	Global
	IEC 62443‐4‐1:2018	Global
	IEC 62443‐4‐2:2019	Global
	UL 2900‐1:2023	Global
	UL 2900‐2‐1:2023	Global
	NIST 800‐53	US
	AAMI SW96:2023	Global
Medical device safety, quality, and risk management	ISO 13485:2016	Global
	UL 2800‐1:2022	Global
	ISO 14971:2019	Global
	IEC 80001‐1:2021	Global
	IEC 80601‐2‐77:2019	Global
Medical device software lifecycle	IEC 62304:2006+A1:2015	Global
Medical device usability	IEC 62366‐1:2015+A1:2020	Global

## 5 Technical Guidelines for Remote Procedures

This section outlines the technical guidelines for the surgical‐grade network, cybersecurity, remote‐enabled robotic system interfaces, telepresence capabilities, and facility‐level requirements for both the remote physician site and patient site to ensure the safe and effective implementation of remote procedure programs.

### 5.1 Surgical‐Grade Network Characteristics

This section outlines the network requirements for safe and effective remote procedures, emphasizing the need for a surgical‐grade network to ensure reliability and maximize uptime. Key characteristics, such as low network latency, minimal jitter, minimal to no packet loss, low error rates, and guaranteed bandwidth, are essential for seamless operation and preventing delays that could compromise patient safety [15, 16, 17]. Additionally, these guidelines highlight the importance of secure communication and data protection to maintain confidentiality and integrity throughout remote procedures [18].

#### 5.1.1 General Surgical‐Grade Network

##### 5.1.1.1 Reliable

1. A surgical‐grade network has the highest level of specifications and shall be optimized for use in remote procedures to ensure reliability, minimize loss of service, and maximize uptime.
2. The surgical‐grade network shall deliver the specified uptime required for the use case.
3. To maximize uptime, the surgical‐grade network shall implement one or more of the following high availability measures:
  - Redundancy with backup systems at various levels (e.g., hardware, connectivity, and power) to prevent single points of failure;
  - Failover mechanisms that automatically switch to backup systems or alternative routes without noticeable delays or service interruptions;
  - Disparate paths to ensure multiple, independent surgical‐grade network routes are available for communication;
  - Software defined surgical‐grade network packet duplication, sending identical data packets via multiple routes to minimize the risk of packet loss during transmission;
  - Other high availability measures.

##### 5.1.1.2 Multipoint Capability

1. The surgical‐grade network shall allow for multipoint capability, such that any given remote physician console can connect to one of multiple patient site robots as deemed necessary by the facilities.
2. The surgical‐grade network shall implement industry‐standard authentication and authorization mechanisms for all users, remote‐enabled robotic systems, and other required connected devices to ensure secure communication and prevent unauthorized access.
3. The surgical‐grade network shall ensure isolation of communications across each site or session, such that there is no interaction of data between separate sites or sessions.
4. The surgical‐grade network may be able to accommodate other related devices or other network services required for the session.

##### 5.1.1.3 Bidirectionality

The surgical‐grade network should deliver consistent, simultaneous, and comparable bandwidth in both directions between the remote physician site and patient site to ensure control and feedback of critical data based on the use case session requirements.

##### 5.1.1.4 Encryption

1. The surgical‐grade network traffic shall be encrypted in transit.
2. Sensitive data shall be encrypted at rest.

##### 5.1.1.5 Session Performance Guarantees

1. When supporting multiple simultaneous sessions, the surgical‐grade network shall maintain the performance and bandwidth of each session.
2. The surgical‐grade network shall be capable of receiving a request to allocate specific bandwidth for a designated block of time and may respond if additional bandwidth is available.
3. The surgical‐grade network shall allocate necessary bandwidth for a session if the requested bandwidth is available. If the requested session is allocated, the total bandwidth required shall be available until the session is ended.
4. The surgical‐grade network shall notify the requestor of the status of the bandwidth allocation.

#### 5.1.2 Quality of Service

##### 5.1.2.1 Network Latency

1. The surgical‐grade network shall provide an indication of network latency to the remote‐enabled robotic system regularly.
2. The surgical‐grade network shall contribute no more than the specified amount of network latency for the use case.
3. The surgical‐grade network shall regularly monitor and report network latency metrics to the remote‐enabled robotic system.

##### 5.1.2.2 jitter

1. The surgical‐grade network shall provide an indication of jitter to the remote‐enabled robotic system regularly.
2. The surgical‐grade network shall contribute no more than the specified amount of jitter for the use case.
3. The surgical‐grade network shall regularly monitor and report jitter statistics to the remote‐enabled robotic system.

##### 5.1.2.3 Packet Loss

1. The surgical‐grade network shall provide an indication of packet loss to the remote‐enabled robotic system regularly.
2. The surgical‐grade network shall contribute no more than the specified amount of packet loss for the use case.
3. The surgical‐grade network shall regularly monitor and report packet loss metrics to the remote‐enabled robotic system.

#### 5.1.3 Bandwidth

##### 5.1.3.1 Sufficient

1. The surgical‐grade network shall guarantee consistent specified bandwidth to carry remote‐enabled robotic system traffic and other required device traffic for authorized sessions.
2. The surgical‐grade network may employ methods, such as QoS/CoS, to maintain consistent bandwidth for any given session.
3. The surgical‐grade network shall provide sufficient capacity for all scheduled sessions, even if conditions require network traffic to switch to an alternative path to reach the intended destination.

##### 5.1.3.2 Scalable

1. The surgical‐grade network shall be capable of receiving and responding to requests from the remote‐enabled robotic system for additional bandwidth during an active session while simultaneously supporting multiple sessions.
2. The bandwidth capacity of the surgical‐grade network shall be capable of accommodating the total peak load bandwidth forecast at any given time.
3. The surgical‐grade network should have a reserved percentage of capacity above and beyond the peak load bandwidth to ensure that unforeseen spikes in utilization and higher than average growth and adoption rates can be accommodated appropriately.

#### 5.1.4 Protocol

1. The surgical‐grade network shall use internet protocol (IP) as its primary transport protocol and adapt as internet protocols evolve.
2. The surgical‐grade network shall use Ethernet (IEEE 802.3) for Layer 2 communications to connect the surgical‐grade network system interface to the remote‐enabled robotic system.

#### 5.1.5 Management

##### 5.1.5.1 Visibility

1. The surgical‐grade network shall track and collect network operational statistics.
2. The surgical‐grade network shall include operational statistics for network latency, jitter, packet loss, and bandwidth.
3. The surgical‐grade network should collect and track other operational statistics and metrics such as bandwidth utilization (e.g., used for specific sessions and devices), device access and authorization, and other session metrics.

##### 5.1.5.2 Real‐Time Status

1. The surgical‐grade network shall convey appropriate real‐time operational status and changes in network conditions to the remote‐enabled robotic system, the remote physician site, patient site care teams, and the network operations team to ensure ongoing safety and security of the remote procedure.
2. Recipients of real‐time surgical‐grade network status updates should interpret the information and respond accordingly.
3. The surgical‐grade network shall deliver updates at a specified frequency, ensuring that recipients can respond effectively.

##### 5.1.5.3 Reporting

1. The surgical‐grade network shall collect required network operational statistics and provide access to these records on a query basis to the remote‐enabled robotic system manufacturer, remote physician site, patient site care teams, and network operations team.
2. Reported surgical‐grade network operational statistics may include network latency, jitter, bandwidth utilization, redundancy status, and packet loss.
3. Remote session logs shall be recorded, retained as required, and accessible to authorized parties.

##### 5.1.5.4 Operational Control

The surgical‐grade network shall effectively manage bandwidth and network traffic while proactively anticipating network utilization to ensure consistency and optimal performance of the session.

### 5.2 Cybersecurity

This section addresses cybersecurity requirements for remote procedures, emphasizing compliance with existing regulations, guidance, and frameworks while addressing unique cybersecurity risks (as e.g., through the exposure to third party and/or public networks) introduced by remote procedures [19, 20, 21, 22, 23]. Rather than relying solely on traditional cybersecurity best practices, this approach identifies cybersecurity gaps and suggests applicable security practices to be considered when a procedure is performed remotely. Beyond reducing the risk of security compromise, these guidelines also prioritize maintaining patient confidence by mitigating concerns about the safety of remote procedures. Best practices from organizations, such as the FDA and NIST are referenced, ensuring a comprehensive and adaptable approach to cybersecurity in remote procedures. See related Sections 4 for cybersecurity standards and (Appendix A2) for a list of general threats that a remote‐enabled robotic system should defend against to address and reduce risks associated with the data and systems used in remote procedures.

#### 5.2.1 Existing Cybersecurity Regulations, Guidance, and Frameworks

1. Existing cybersecurity regulations shall be followed as applicable.
2. Existing cybersecurity guidance and frameworks should be followed as applicable.
3. Cybersecurity regulations, guidance, and frameworks that may be applicable to remote procedures include, but are not limited to, the following examples shown in Table 2.

**TABLE 2:** Examples of existing cybersecurity regulations, guidance, and frameworks that may be applicable to remote procedures (see Table 1 for cybersecurity standards).

Category	Regulations, guidance, and frameworks
Regional market approval	FDA Cybersecurity in Medical Devices: Quality System Considerations and Content of Premarket Submissions (2023)
	FDA Postmarket Management of Cybersecurity in Medical Devices (2016)
	FDA 21 CFR Part 820
	HIPAA 45 CFR Part 160, Part 164
	EU GDPR
	EU MDR
Global development	IMDR/CYBER WG/N70FINAL:2023
	NIST SP 800‐131A Rev. 2 (2019)
	IETF TLS 1.3 Protocol
	ISO/IEEE 11073‐40101:2022
	ISO/IEEE 11073‐40102:2022
	FIPS 140‐3
Premarket risk	FDA CFR 820.50
	AAMI TIR 57:2016
Cybersecurity	AAMI TIR 97:2019
	NIST 800‐161r1
	IETF TLS 1.3 Protocol

#### 5.2.2 Guidelines for Cybersecurity

##### 5.2.2.1 Introduction of a Third‐Party Surgical‐Grade Network Provider

1. The remote‐enabled robotic system manufacturer shall design the remote‐enabled robotic system for defense in depth and assume the surgical‐grade network infrastructure between the remote physician console and the patient site robot is hostile and may, at any given time, experience compromise or disruption.
2. The remote‐enabled robotic system manufacturer shall implement the necessary security controls to ensure the confidentiality, integrity, and availability of data traveling between the remote physician console and the patient site robot.
3. The remote‐enabled robotic system manufacturer shall integrate failsafe mechanisms and safety controls to protect the patient, users, property, and the environment from harm.
4. The surgical‐grade network provider shall implement the required security and operational controls to ensure that network performance, uptime, and QoS metrics required for remote procedures are not adversely impacted by either malicious actors or systemic failures. These controls shall address:
  - Confidentiality protections (e.g., segregation of all remote‐enabled robotic system sessions);
  - Integrity protections (e.g., using Message Authentication Codes to verify that the data has not been altered during transmission);
  - Availability (e.g., protection against and response to DDoS attacks, bandwidth congestion, and infrastructure failures);
  - Performance and Reliability (e.g., network latency minimization, jitter control, and real‐time failover);
  - Disaster Recovery and Business Continuity (e.g., redundancy, geo‐distributed backups, and rapid recovery).

##### 5.2.2.2 Multitenant Nature of the Surgical‐Grade Network

1. The remote‐enabled robotic system shall employ best practices to mutually authenticate connections and communications between its remote‐enabled robotic subsystems to prevent unintended communications.
2. The remote‐enabled robotic system manufacturer and the surgical‐grade network shall implement end‐to‐end encryption between all network nodes.
3. The remote‐enabled robotic system shall implement end‐to‐end encryption between all endpoints on the surgical‐grade network.
4. The remote‐enabled robotic system shall employ encrypted protocols from the remote physician site console and patient site robot to any and all external network services.
5. The remote‐enabled robotic system manufacturer should implement additional data privacy protections to protect personal and sensitive data, considering that hospital‐based security controls commonly implemented to keep data private may not be present on the third‐party providers' surgical‐grade network.
6. The remote‐enabled robotic system manufacturer, and where applicable the surgical‐grade network provider, shall ensure that steps are taken to isolate each site/system/user's infrastructure and equipment, including the remote‐enabled robotic system from untrusted connections by other systems or unauthorized access by other users.

##### 5.2.2.3 Lack of Physical Proximity Between the Surgeon and Patient

1. The remote‐enabled robotic system manufacturer, in coordination with the surgical‐grade network provider, shall implement security controls that ensure the correct components (e.g., remote physician console and patient site remote‐enabled robot) are being paired.
2. The remote‐enabled robotic system manufacturer may make use of a shared “session key” that is communicated between the remote physician and the patient‐site team and shared “out of band” (e.g., an alpha and/or numeric code that needs to be verbally communicated between the parties). The correct session key shall be required to initiate communication between the remote physician console and the patient site robots.

##### 5.2.2.4 Potential Introduction of Adverse Effects Through Security Controls

1. The remote‐enabled robotic system's security controls shall be evaluated to ensure that they do not lead to an uncontrolled or undesired change in system behavior (e.g., undesirable additional teleoperation latency, jitter, or worst‐case unexpected movement).
2. The remote‐enabled robotic system manufacturer's design shall include measures to reduce the risk of security failures as well as mechanisms for handling errors and exceptions. Any failures shall be addressed through fail‐safe features, ensuring that they do not cause undesirable system behavior or disrupt an ongoing procedure.

##### 5.2.2.5 Robust and Secure Surgical‐Grade Network Operations

The network operations team should evaluate industry best practices for cybersecurity, including but not limited to the following:

1. Implement surgical‐grade network security tools to aid with the detection (e.g., NDR, Network Detection and Response or NIDS, Network Intrusion Detection System) as well as Security Incident and Event Monitoring (SIEM) to collectively enable the timely detection, analysis, and response to anomalous traffic and events;
2. Implement surgical‐grade network and security log retention capability to meet regulatory and operational requirements;
3. Implement node‐level security protections such as Endpoint Detection and Response (EDR) or equivalent protections;
4. Implement a policy and procedure for risks related to supply‐chain, subcontractor, and third party partners/vendors;
5. Conduct regular cybersecurity penetration testing, preferably through a well‐recognized neutral third party testing provider;
6. Conduct regular vulnerability scanning and monitoring of the network and connected infrastructure;
7. Develop and maintain an asset database and incident response policy and plan;
8. Implement a regular and recurring security review process that addresses logs and events, changes to asset database(s), access provisioning including changes/adds/deletes/stale‐records, and least‐privilege requirements.

### 5.3 Remote‐Enabled Robotic System Interfaces

This section addresses the critical interfaces between the remote‐enabled robotic system and the surgical‐grade network, which are essential for safe and effective remote procedures. It outlines the necessary functions required for connectivity and operation, ensuring reliable communication, secure operation, and efficient management of remote robotic procedures. See related Section (Appendix A1) for a list of other remote‐enabled robotic system considerations.

#### 5.3.1 Functions for Remote‐Enabled Robotic Systems to Connect and Operate Over a Surgical‐Grade Network

##### 5.3.1.1 Device ID and System Information

1. Each device connected to the surgical‐grade network shall have a guid that is communicated to the network for connectivity management and security.
2. All guids shall be cryptographically protected and verifiable through strong cryptographic algorithms.

##### 5.3.1.2 Data Encryption, Decryption

remote‐enabled robotic system traffic shall be encrypted at the application layer. (Refer to Section 5.2.2.2.)

##### 5.3.1.3 Data Packetization

Each device connected to the surgical‐grade network shall be able to interface with a standard layer 1–3 network and support pmtud to prevent fragmentation and optimize data transfer.

##### 5.3.1.4 Ports

1. Proprietary communication protocols shall use nonwell‐known ports.
2. Standard communication protocols shall use standard ports.

##### 5.3.1.5 Protocols

1. Each device application should communicate using either the TCP or UDP protocol.
2. Devices should use standard security communication protocols and avoid protocols that are considered insecure (e.g., use SSH instead of Telnet, HTTPS instead of HTTP, and TLS 1.2/1.3 with strong cipher suites instead of TLS 1.1).
3. Device applications shall use protocols that can be forwarded across a layer‐3 IP network.

##### 5.3.1.6 Routing and IP Addressing

The surgical‐grade network interface for each device shall have a unique IP address for each LAN within a specified remote‐enabled robotic system.

##### 5.3.1.7 Ability to React to Changes in Surgical‐Grade Network Conditions

1. The remote‐enabled robotic system shall notify users of appropriate status changes in surgical‐grade network conditions.
2. The remote‐enabled robotic system and users shall interpret and respond appropriately to status changes in surgical‐grade network conditions to ensure safety.

##### 5.3.1.8 Device Surgical‐Grade Network Authentication

Connected devices shall authenticate to the surgical‐grade network using industry‐recognized standard authentication methods.

##### 5.3.1.9 Session Start‐Up Sequence

1. A surgical‐grade network connection to initiate a session shall only be established if the remote‐enabled robotic system indicates that the surgical‐grade network QoS requirements can be met for the use case.
2. The remote physician site console and the patient site robot may power up independently and asynchronously.

##### 5.3.1.10 Session End Sequence

The remote‐enabled robotic system shall notify the surgical‐grade network when the session has been completed, and the surgical‐grade network shall acknowledge receipt of the notification.

##### 5.3.1.11 Disconnected Session

If the surgical‐grade network stops detecting communication between the remote physician console and the patient site robots, the network shall notify the remote‐enabled robotic system that no robot‐system communication is being detected and the remote‐enabled robotic system should end the session.

##### 5.3.1.12 Remote‐Enabled Robotic System Onboarding

1. The remote‐enabled robotic system manufacturer shall authorize specific models or configurations of remote‐enabled robotic systems, including the bandwidth requirements of that configuration, to connect over the surgical‐grade network.
2. The healthcare system shall authorize specific remote‐enabled robotic system devices of an authorized model or configuration to connect with each other over the surgical‐grade network.
3. The specific authorized models or configurations of remote‐enabled robotic system devices shall be communicated to the surgical‐grade network.

##### 5.3.1.13 Remote‐Enabled Robotic System Offboarding

1. remote‐enabled robotic system operators may deauthorize a specific model or configuration of a remote‐enabled robotic system from operating over the surgical‐grade network.
2. All specific remote‐enabled robotic system devices in a de‐authorized model or configuration shall be deauthorized from operating over the surgical‐grade network.
3. The healthcare system may deauthorize a specific model or configuration of a remote‐enabled robotic system from operating over the surgical‐grade network.
4. The specific models or configurations of deauthorized remote‐enabled robotic system devices shall be communicated to the surgical‐grade network.

#### 5.3.2 Required Data Exchange for Remote‐Enabled Robotic Systems to Operate Over a Surgical‐Grade Network

##### 5.3.2.1 Surgical‐Grade Network Status and Health

The surgical‐grade network shall supply the remote‐enabled robotic system and other components with performance status and health statistics to ensure confidence in performance and facilitate risk assessment.

##### 5.3.2.2 Fleet Status

The remote‐enabled robotic system and other required devices shall report performance status and health statistics to the surgical‐grade network to enable readiness for the planned session.

##### 5.3.2.3 Session Logs

network performance logs for each session shall be recorded, retained as required, and accessible to authorized parties for forensic analysis, troubleshooting, and other purposes.

### 5.4 Telepresence

This section addresses the critical role of telepresence in remote procedures. It emphasizes the necessity of high‐quality real‐time network communications consisting of two‐way video and audio between the remote physician site and the patient site procedure room. These requirements are essential for maintaining situational awareness, ensuring patient safety, and facilitating a seamless workflow when the remote physician and patient are physically separated [24].

#### 5.4.1 Video in Remote Physician Site and Patient Site Procedure Rooms

1. The remote physician site shall have an adequate view of the patient, other participants, and critical monitors and displays at the patient site via telepresence to ensure safety and facilitate seamless workflow.
2. The room‐to‐room video connection between the remote physician site and the patient site procedure room shall be transmitted over a surgical‐grade network or a network of equivalent reliability as defined in Section 5.1.1.1.
3. The remote physician site should have the capability to access multiple room views as needed for the use case.
4. The remote physician site should have the pan, tilt, and zoom capabilities for optimal visualization of the patient site monitors and displays as needed per the use case.
5. Participants in the patient site procedure room should have an adequate view of the remote physician.
6. The remote physician and care team members may be notified if the room‐to‐room video connection is lost during a session.

#### 5.4.2 Audio in Remote Physician Site and Patient Site Procedure Rooms

1. The remote physician site shall have the capability of real‐time network communications consisting of two‐way audio, with participants in the patient site procedure room to maintain situational awareness, ensure safety, and facilitate seamless workflow.
2. The remote physician site shall be able to hear critical equipment sounds, including electrocautery signal settings, activation indicators, and fault alerts, within the required timeframe.
3. Audio communications between the remote physician site and patient site participants shall be transmitted over a surgical‐grade network or a network of equivalent reliability as defined in Section 5.1.1.1
4. The remote physician and care team members shall be notified if audio communication is lost during a session.

### 5.5 Remote Physician Site and Patient Site Facilities

1. The remote physician site and patient site shall ensure the remote‐enabled robotic system is authorized for clinical use in remote procedures by the relevant authority (e.g., the FDA).
2. The remote physician site and patient site shall comply with all applicable federal, state, and international laws, regulations, guidelines, and standards.
3. The remote physician site and patient site shall establish comprehensive plans and policies for information privacy and security compliance in remote procedures and ensure that contracts or agreements for remote procedure support include specific security controls for information security management.
4. The remote physician site and patient site shall have access to appropriately trained and qualified technical support personnel (e.g., clinical engineers) with knowledge and skills in the maintenance and management of remote‐enabled robotic systems and remote procedures (e.g., network troubleshooting and fail‐safe drills).
5. The remote physician site and patient site shall ensure proper performance validation testing and maintenance of the remote‐enabled robotic system infrastructure in accordance with relevant policies and procedures of the relevant governing organizations, manufacturer guidelines, and other industry best practices to ensure the remote‐enabled robotic system remains safe, effective, and compliant with regulatory requirements.
6. The remote physician site and patient site shall ensure the appropriate surgical‐grade network infrastructure required for the remote procedure.
7. The patient site for a remote procedure shall meet the same space requirements as local procedures and comply with all relevant standards and regulations to ensure safety, sterility, and functionality of the surgical environment.
8. The remote physician site shall be a dedicated space equipped with adequate power, ensuring safety, privacy, and comfort.

## Author Contributions

**Yulun Wang:** conceptualization, writing – original draft, writing – review and editing. **Martin Buehler:** writing – review and editing. **Shane Farritor:** writing – review and editing. **Yuman Fong:** writing – review and editing. **Mike Kijewski:** writing – review and editing. **Brian Miller:** writing – review and editing. **Bill Peine:** writing – review and editing. **Blair Whitney:** conceptualization, writing – original draft, writing – review and editing. **Mike Yramategui:** writing – review and editing. **Jordana Bernard:** conceptualization, writing – original draft, writing – review and editing, project administration.

## Conflicts of Interest

Yulun Wang declares Teladoc Fellow, Cottage Health Board Member, World Telehealth Initiative Chairman and Cofounder, stock options Sovato; Martin Buehler declares stock options Johnson & Johnson; Shane Farritor declares stock and patents Virtual Incision; Yuman Fong declares scientific consultant for Medtronic, Johnson & Johnson, and Imugene, receives royalties from Merck and Imugene, and owns patents for CF33‐OVs and CF33‐CD19t licensed to Imugene LTD; Mike Kijewski declares none; Brian Miller declares stock options Intuitive Surgical; Bill Peine declares stock, stock options, patents, and grant funding from Office of Naval Research, Grant 14143260, SC 1000020614, “Advanced Tele‐Robotic Surgery for Combat Casualty Care;” Blair Whitney declares stock options Sovato; Mike Yramategui declares stock options Intuitive Surgical; and Jordana Bernard declares stock options Sovato.

## Data Availability

The authors have nothing to report.

## Appendix A

### A1 Other Remote‐Enabled Robotic System Considerations

This section outlines additional technical design and implementation considerations for remote‐enabled robotic systems to ensure the safety and effectiveness of remote procedures beyond the core guidelines discussed in previous sections. These considerations highlight important factors for adapting existing robotic systems to function remotely and address specific challenges introduced by network‐dependent operations.

1. Ensuring teleoperation latency, which will vary by remote‐enabled robotic system and distance between the remote physician site and patient site, is acceptable for a given use case.
2. Ensuring the remote‐enabled robotic system can tolerate (per the SLA of the surgical‐grade network) surgical‐grade network properties, such as network latency, jitter, and packet loss. Ensuring the remote‐enabled robotic system can reestablish a connection after a session timeout. Designing system acknowledgments, such as heartbeats and command statuses, to prevent timeouts given the teleoperation latency. Testing the system at maximum teleoperation latency to confirm the system's ability to handle potential timeouts effectively.
3. Ensuring that the remote‐enabled robotic system can adjust parameters based on dynamic surgical‐grade network conditions. Adaptability to changing surgical‐grade network conditions while maintaining performance. The remote‐enabled robotic system notifying the remote physician of degrading surgical‐grade network conditions that may affect performance.
4. Ensuring the start‐up sequence of the remote‐enabled robotic system accounts for the separation between the remote console and the rest of the system. Designing the remote console and the patient site robot to boot up independently, ensuring seamless operation despite their physical separation. Integrating this independent startup process into the system's design to accommodate surgical‐grade network constraints and maintain functionality.
5. Utilizing a low‐loss low‐latency codec for endoscopic video or imaging, as it serves as the primary feedback mechanism for remote procedures. This approach preserves high‐quality visuals, ensuring that video quality is indistinguishable from that of same‐room imaging.
6. Ensuring energy source control remains reliable and tolerant of communication degradation and failures. Implementing a fail‐safe design and clear indications of the device status and settings to enable effective awareness for the remote user.
7. Ensuring patient site clinicians have the capability of halting the remote site physician's control of the remote‐enabled robot when necessary, serving as a critical safety mechanism.
8. Allowing both the remote physician site and patient site to pause or temporarily interrupt instrument control during the procedure when necessary, enhancing overall safety.
9. Ensuring a safe, seamless, and definitive transition of control between a remote console and another console.
10. The remote‐enabled robotic system manufacturer ensures controls and visible warnings are implemented to prevent physical injury when the patient site staff enter the movement safety zone of articulating components during a remote procedure.
11. The remote‐enabled robotic system manufacturer implements a method for the patient site staff to quickly pause remote movement control, bidirectional audio–video; and terminate a remote session without automatic remote reconnection.

### A2 General Threats a Remote‐Enabled Robotic System Should Defend Against

In addition to the cybersecurity gaps covered in Section 5.2, developers of remote‐enabled robotic systems should adhere to established best practices for identifying, enumerating, and defining requirements for cybersecurity risks. Specifically, threat modeling must be performed to analyze and mitigate risks associated with both the overall remote‐enabled robotic system architecture and its individual components [25, 26]. Additionally, the following key security practices address and reduce risks to the confidentiality, integrity, and availability of the data and systems, regardless of their remote capabilities:

1. Compliance with generally accepted security requirements and security engineering best practices.
2. Implementation of plans and processes for cybersecurity premarket and postmarket risk management, including identification, communication, and mitigation of new risks as well as securing and distributing software updates and patches.
3. Follow generally recognized security principles (e.g., Secure by Design, Secure by Default, Least Privilege, and Defense in Depth).
4. Follow best practices for software security review and analysis, including threat modeling, attack surface analysis, software composition analysis, code review, and SBOM vulnerability analysis.
5. Follow best practices for software testing, including robustness testing, static application security testing (SAST), dynamic application security testing (DAST), boundary value analysis, fuzz testing, vulnerability scanning, and penetration testing.
6. Provide evidence of testing at the respective integration stages and for the total system, including traceability of security requirements and identified risks to test results.
7. Formalize and document any decisions regarding risks resulting from identified vulnerabilities that could not be or were not mitigated.
8. Formalize and document any security trade‐off decisions (e.g., security vs. safety, security vs. usability, and security vs. features).
9. Provide documentary evidence supporting the above security practices.
10. User instructions and diagrams pertaining to cybersecurity, such as secure installation and integration, surgical‐grade network requirements, security infrastructure requirements, and security incident response handling.
  Remote procedures, by definition, introduce a data path that sends command and control data for remote‐enabled robotic systems outside the hospital network. This data path introduces the potential for heightened risks. Some related threats to consider include:
11. Attacks on external surgical‐grade network infrastructure, impeding the availability and real‐time capabilities of these systems.
12. A man‐in‐the‐middle attack on an external data center.
13. Compromise of personal or sensitive data as it leaves the hospital's network.

Networks used for remote procedures should be designed with threats, such as these in mind, and built with mitigation strategies to anticipate these risks.

(See Section 5.2 for cybersecurity guidelines and requirements for remote procedures).
